# Intrauterine device migration into the lumen of large bowel: A case report

**DOI:** 10.1016/j.ijscr.2020.06.011

**Published:** 2020-06-12

**Authors:** Vygintas Aliukonis, Marius Lasinskas, Algirdas Pilvelis, Audrius Gradauskas

**Affiliations:** a Department of Abdominal Surgery, Clinic of Surgery, Vilnius City Clinical Hospital, Antakalnio 57, LT-10207, Vilnius, Lithuania; b Centre for Health Ethics, Law and History, Institute of Health Sciences, Faculty of Medicine, Vilnius University, 03101 Vilnius, Lithuania; c Department of Nursing and Fundamentals of Internal Medicine, Faculty of Medicine, Vilnius University, Antakalnio 57, LT-10207, Vilnius, Lithuania

**Keywords:** Perforation, Intrauterine device, Colon, Laparoscopy, Migration, Missing IUD

## Abstract

**Introduction:**

Colon perforation caused by the intrauterine device (IUD) migration is rare, but severe complication that can occur years after the insertion. Depending on the location of the injured intestine, the different methods for extracting migrated IUD raging from colonoscopy to laparotomy can be chosen.

**Case presentation:**

A 41-year-old female presented with the discomfort in the epigastric area went to the outpatient clinic. A doctor performed gastroscopy and found a polyp. During a colonoscopy, we found a small solid object protruding through the intestinal wall. CT scan showed IUD like “T” shape foreign body, which longest part was protruding a wall of the colon. After these CT findings, the patient informed us that she had IUD inserted almost ten years ago. We found the device with the laparoscopic approach. IUD was removed and the intestinal damage was repaired during the mini-laparotomy.

**Conclusions:**

Perforation of IUD can be asymptomatic, although sometimes it can cause short-term or long-term symptoms. Penetrated IUDs should be removed whenever identified. For intra-abdominal penetrations, the laparoscopic or mini-laparotomy approach is a safe and appropriate method.

## Highlights

• Colon perforation caused by Intrauterine Device is a rare but severe complication.
• No similar cases were found after reviewing articles in over a 10-year period.
• It is still recommended to remove any free foreign body in abdominal cavity.
• For intraabdominal penetrations, the laparoscopic approach is an appropriate method.

## 1 Introduction

The first modern intrauterine device (IUD) was introduced as early as 1909 [1]. Since then, IUD has become one of the world’s most popular and modern means for contraception [2].

IUD is usually placed without any significant complication. However, as with any intervention, there are several possible drawbacks. For instance, hemorrhage, infection, migration, rupture, dislocation, and downward movement. The one that gets the most attention is perforation. Uterine perforation by IUD is reported between 0.5 and 13 per 1000 insertions [3,4].

Most uterine perforations do not influence other organs. Regardless of that, 15% of cases leads to the complications in the adjacent organs, especially in terms of the intestine. The intestinal complications associated with IUD migration are the followings: obstruction, infarction, fistula formation, mesenteric injury, and perforation. IUD intestinal penetration in large part occurs in the sigmoid colon (40.4%), small intestine (21.3%), and rectum (21.3%)) [5,6].

Higher rates for the complications are associated with the inexperience of the inserter, post-partum status, breastfeeding status, and abnormal uterine cavity anatomy [7].

In this article, we will look into IUD migration to the splenic flexure of the large bowel.

This work is in line with the SCARE criteria [8].

## 2 Case report

A 41-year-old female presented with the discomfort in the epigastric area went to the outpatient clinic. The gastroscopy was performed and the polyp was found. The histologic study showed no malignancy. When a polyp was found in a stomach, then the colonoscopy was ordered.

**Fig. 1:**
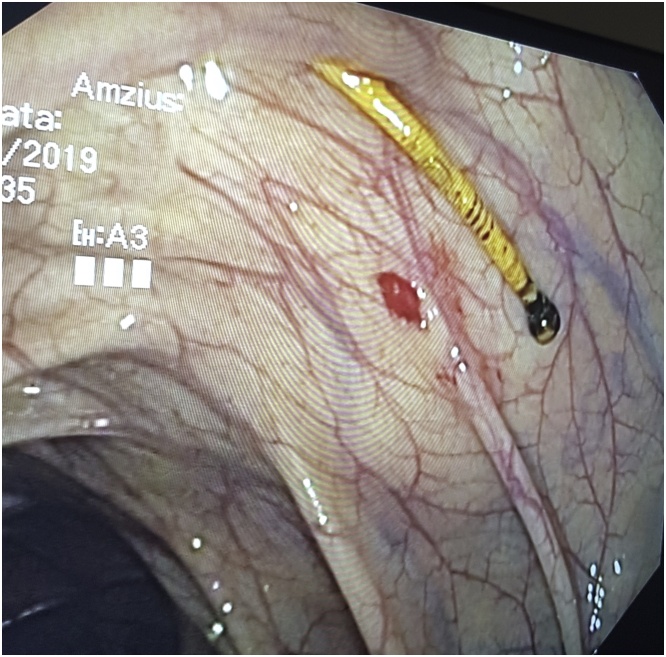
Object protruding through the intestinal wall.

**Fig. 2:**
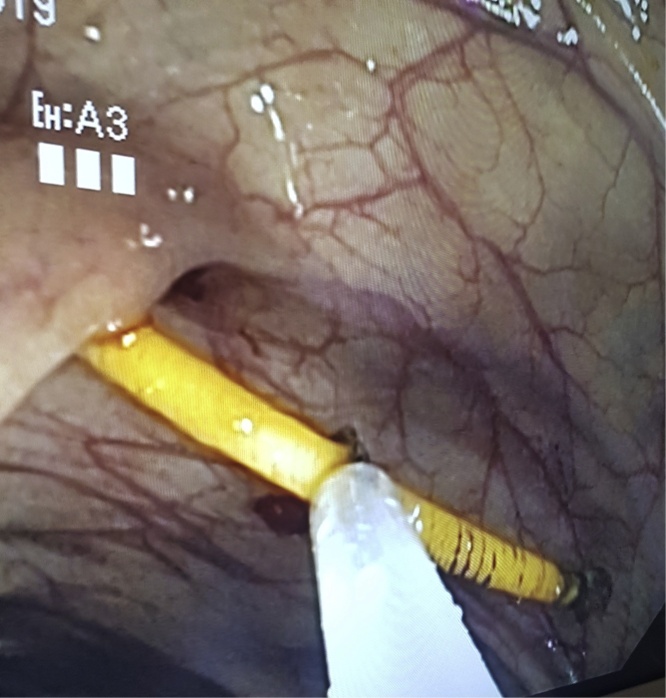
Attempt to pull out object with endoscopic loop.

During the examination, a small solid object was observed protruding through the intestinal wall (Fig. 1). We attempted to pull it out with an endoscopic loop (Fig. 2). However, it was firmly adherent to the colon wall. In order not to cause any damage to the intestine, we decided to discontinue our efforts. The rest of the colonoscopy was eventless.

**Fig. 3:**
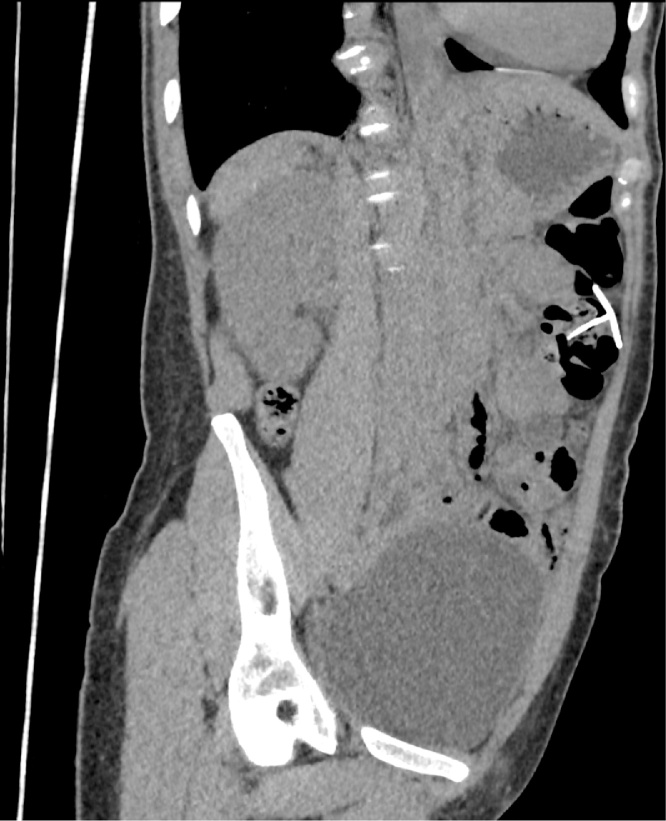
CT with a foreign body.

CT scan showed an IUD like “T” shape foreign body (Fig. 3). The longest part was protruding through the wall of the splenic flexure of the colon and the transversal part was in the abdominal cavity.

After these CT findings, the patient informed us that she had IUD inserted almost ten years ago. Two years after the procedure, she gave birth via natural way. The patient admitted that she did not seek any medical advice concerning IUD, because she thought it fell out.

**Fig. 4:**
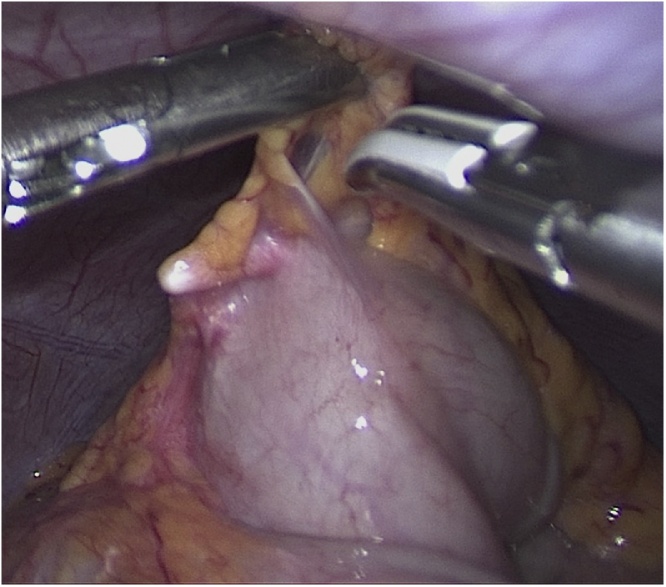
A foreign body during laparoscopy.

The device was found at the location of the splenic flexure with a laparoscopic approach (Fig. 4). The longest part of IUD was the penetrating wall of the colon. Three centimeters width mini-laparotomy was done in the upper left quadrant, IUD was removed, and the intestinal damage was repaired.

### 2.1 Postoperative period

The postoperative period was eventless. The patient was discharged from the hospital after three days and the treatment continued on an outpatient basis.

## 3 Discussion

The literature search was conducted in PubMed on the 15th of December 2019, using the search terms “intrauterine device” and “perforation”, covering the last ten years. The search was limited to peer-review articles published in English. There was in total 53 matches. After removing duplicating and non-colon related issues, 22 articles dealing with the colon perforation were included in our analysis.

Study by Rao et al. shown that almost two-thirds of lost IUD are generally located inside the uterine cavity [9]. However, according to the study made by Cetinkaya et al. most common extra-uterine location of lost IUDs is around the uterosacral ligaments [10].

We present our case because it is extremely rare. No similar cases were found after reviewing articles in over a period of ten years. This is most likely associated with a particular location of the perforation – the splenic flexure is relatively distant from the uterus.

A possible mechanism for how migration could occur is uterine enlargement during the patient’s pregnancy. However, as stated by Takahashi et al., there is no past literature associating pregnancy as a risk factor for extrauterine IUD organ penetrationx [11].

In a majority of cases associated with IUD migration, the patients do not express any symptoms and the device can remain there for many years [12,13].

However, if there are no comorbidities, it is still recommended to remove any free foreign body in the abdominal cavity due to the possible adhesion formation that may cause small bowel obstruction or possible injuries to the adjacent organs [14].

Depending on the location, serious complications occur in about 15 % of cases of IUD perforations [15].

In order to have a better diagnosis, the most common exact anatomical location and visceral relationships can then be demonstrated by a computed tomography scan [16].

According to the injury site of intestine there are different methods for extracting migrated IUDs ranging from colonoscopy to laparotomy [17]. Due to the rarity of cases like these, the preferred treatment is left for the surgeon to choose.

Recent article by Lei et al. suggests that endoscopic rather than the surgical removal of IUDs should be considered if injury is located in the sigmoid colon and distally. Endoscopy is safe, efficient, and cost-effective strategy [18].

However, when the injury associated location is in the proximal part of the colon, the endoscopic extraction might be too unpredictable and involves unnecessary risk.

A systematic review by Gill et al. showed that the success rate of laparoscopy was almost two-thirds (64%) whereas success of mini-laparotomy was significantly higher (94%) [19].

## 4 Conclusion

Intrauterine devices are known as a commonly used and safe contraceptive method, nevertheless, various complications of this method can occur. The patients with the abdominal complaints and history of having IUD that is missing at the present should be considered as the patients with possible IUD migration. Ultrasound and computed tomography are the first choices for locating missing IUD. Penetrated IUDs should be removed whenever identified. The laparoscopic or mini-laparotomy approach is a safe and appropriate method for the intra-abdominal penetrations.

## Declaration of Competing Interest

The authors have no conflict of interest to disclose.

## Funding

This research did not receive any specific grant from funding agencies in the public, commercial, or not-for-profit sectors.

## Ethical approval

Case reports with written patient consent do not require ethical approval in Lithuania.

## Consent

The patient allowed us to use her information and signed the written consent.

## Author contribution

All authors were involved in writing - original draft ; writing - review & editing ; conceptualization.

## Registration of research studies

NA.

## Guarantor

Vygintas Aliukonis.

## Provenance and peer review

Not commissioned, externally peer-reviewed.
